# Widely metastatic glioblastoma with *BRCA1* and *ARID1A* mutations: a case report

**DOI:** 10.1186/s12885-020-6540-1

**Published:** 2020-01-20

**Authors:** Melissa Umphlett, Stephanie Shea, Jessica Tome-Garcia, Yizhou Zhang, Adilia Hormigo, Mary Fowkes, Nadejda M. Tsankova, Raymund L. Yong

**Affiliations:** 1 Department of Pathology, Icahn School of Medicine at Mount Sinai, New York, NY USA; 2 Departments of Neurosurgery and Oncological Sciences, Icahn School of Medicine at Mount Sinai, New York, NY USA; 3 Department of Neurology, Medicine (Division Hem-Onc), Neurosurgery and the Tisch Cancer Institute, Icahn School of Medicine at Mount Sinai, New York, NY USA; 4 Department of Neuroscience, Icahn School of Medicine at Mount Sinai, New York, NY USA

**Keywords:** Glioblastoma, Metastasis, BRCA1 mutation, ARID1A mutation, Mismatch repair deficiency, Temozolomide, Treatment resistance

## Abstract

**Background:**

Glioblastoma (GBM) is a highly malignant brain neoplasm with poor survival. Despite its aggressive nature, metastatic spread of GBM is identified only rarely. While the molecular alterations associated with GBM and its subtypes are well-described, there remains a gap in understanding which alterations may predispose towards metastasis. In this report, we present a case of GBM with multi-organ metastases and discuss its genomic alterations.

**Case presentation:**

A 74-year-old woman was diagnosed with left occipital glioblastoma (IDH-wildtype, MGMT-unmethylated), for which she underwent resection, standard chemoradiation, and then stereotactic radiosurgery (SRS) for local recurrence. One month after SRS, work-up for a pathologic hip fracture revealed a left breast mass, lytic lesions involving pelvic bones, and multiple pulmonary and hepatic lesions. Biopsies of the breast and bone lesions both demonstrated metastatic IDH-wildtype GBM. For worsening neurologic symptoms, the patient underwent debulking of a large right temporal lobe recurrence and expired shortly thereafter. Autopsy confirmed metastatic GBM in multiple systemic sites, including bilateral lungs, heart, liver, thyroid, left breast, small bowel, omentum, peritoneal surfaces, visceral surfaces, left pelvic bone, and hilar lymph nodes. Targeted sequencing was performed on tissue samples obtained pre- and postmortem, as well as on cell cultures and an orthotopic mouse xenograft derived from premortem surgical specimens. A *BRCA1* mutation (p.I571T) was the only variant found in common among the primary, recurrence, and metastatic specimens, suggesting its likely status as an early driver mutation. Multiple subclonal *ARID1A* mutations, which promote genomic instability through impairment of DNA mismatch repair, were identified only in the recurrence. Mutational spectrum analysis demonstrated a high percentage of C:G to T:A transitions in the post-treatment samples but not in the primary tumor.

**Conclusion:**

This case report examines a rare case of widely metastatic IDH-wildtype GBM with a clonal somatic mutation in *BRCA1*. Post-treatment recurrent tumor in the brain and in multiple systemic organs exhibited evidence of acquired DNA mismatch repair deficiency, which may be explained by functional loss of *ARID1A*. We identify a potential role for immune checkpoint and PARP inhibitors in the treatment of metastatic GBM.

## Background

Glioblastoma (GBM) is the most common primary brain tumor in adults and universally harbors a poor prognosis due to its aggressive nature [1]. Despite modern improvements in treatment for afflicted patients, the mortality of GBM remains high, with a median overall survival of 10–16.5 months [2]. Although it is commonly associated with widespread infiltration throughout the brain, GBM is only rarely associated with extracranial metastatic disease [3, 4], which occurs at an estimated incidence of less than 2% [5–11]. Widespread multi-organ metastases are rarer still. A literature review of 79 cases of extracranial metastatic GBM found that only 4% of cases examined had greater than four metastatic sites [12]. Furthermore, to our knowledge there are only nine reported cases of high-grade glioma metastases involving skin, soft tissue, or muscle [13].

Possible explanations for the rarity of GBM systemic metastases include underdiagnosis and short patient survival time [14]. Case reports have described the diagnosis of metastatic GBM in recipients of lung, liver, and other organ transplants from deceased donors harboring GBM, indicating that GBM micrometastases may be present at the time of death [14, 15]. Such cases suggest that rates of clinically detected GBM metastases may underestimate the extent to which these malignant tumors are capable of seeding distant organs. The underlying genomic drivers of systemic GBM metastases remain poorly defined. Limited molecular analyses of several reported cases have suggested an association with mutations in *TP53* [16]; however, *TP53* mutations are also among the most common across all cancer.

Recognizing the importance of identifying unique molecular features that may drive extracranial GBM metastasis, we present a rare case with widespread multi-organ metastases, placing special attention on a comparative analysis of the most frequent genetic alterations found in the primary tumor, its post-treatment brain recurrence, and multiple systemic metastatic sites.

## Case presentation

**Fig. 1:**
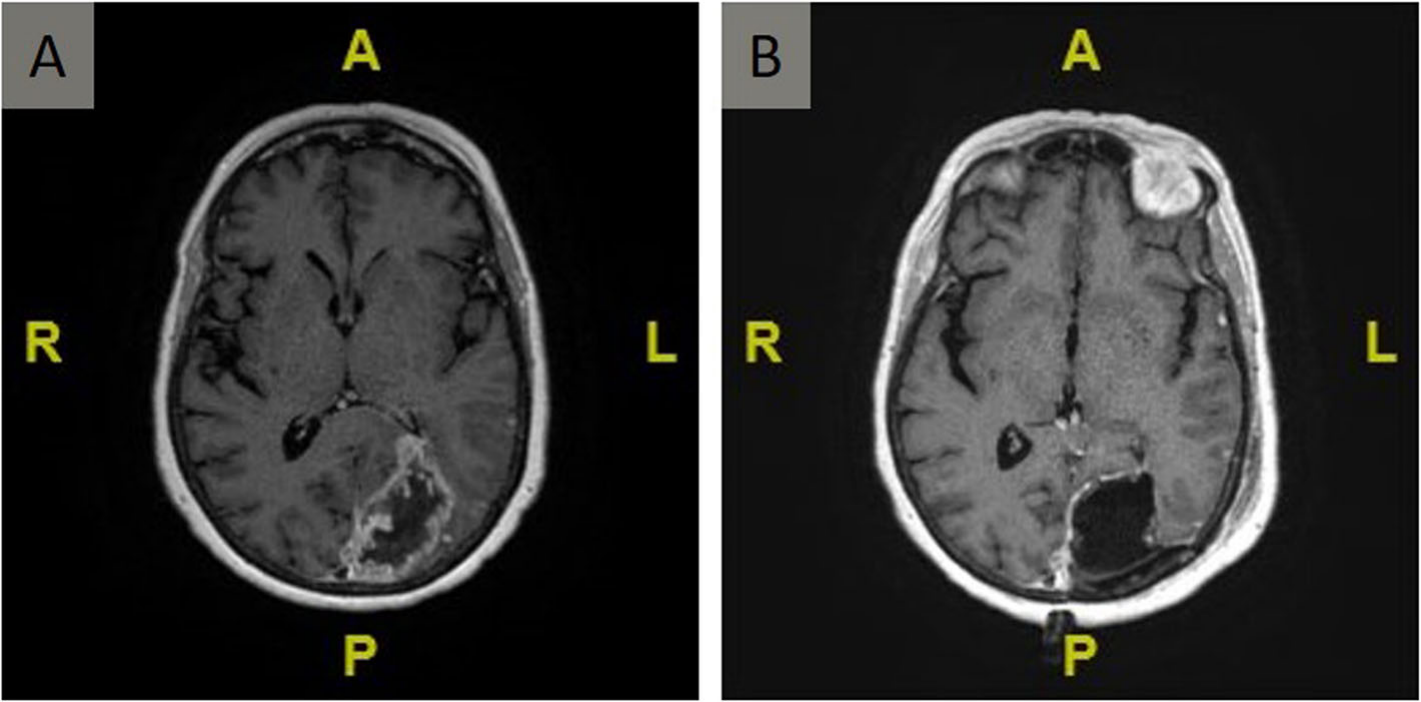
Primary GBM in the left occipital lobe. Axial T1 gadolinium-enhanced magnetic resonance image performed (**a**) preoperatively, and (**b**) 24 h postoperatively

A 74-year-old female was initially evaluated for a headache and right eye peripheral vision loss. MRI brain with and without contrast was performed, revealing a 5.5 cm heterogeneously T2 hyperintense lesion with thick irregular nodular enhancement in the left parietal-occipital region (Fig. 1). The patient underwent a gross total resection of the mass that was diagnosed as GBM, IDH-wildtype, WHO grade IV, MGMT promoter methylation not detected. Fresh specimen in multiple sectors was processed for tumor culture. Following resection, she received hypofractionated concurrent chemoradiation with temozolomide followed by four cycles of adjuvant temozolomide (TMZ). Six months later, the patient developed multifocal GBM recurrence in the right temporal and frontal lobes, for which she underwent single fraction 18 Gy stereotactic radiosurgery to the right frontal lesion and five fractions of 2250 cGy to the right temporal lesion.

Three months following SRS, the patient began to experience falls associated with hip pain and difficulty walking. MRI of the right hip demonstrated a pathologic hip fracture, which was thought to be due to metastatic disease from an undiagnosed second primary cancer. The patient then developed altered mental status and right-sided upper motor neuron facial weakness. A full metastatic imaging work-up was performed, revealing a 3.9 cm left breast mass, multiple lytic lesions of the pelvic bones, and multiple pulmonary and hepatic nodules. Core biopsies were obtained from the left breast and the left pubic bone, both of which demonstrated metastatic GBM.

**Fig. 2:**
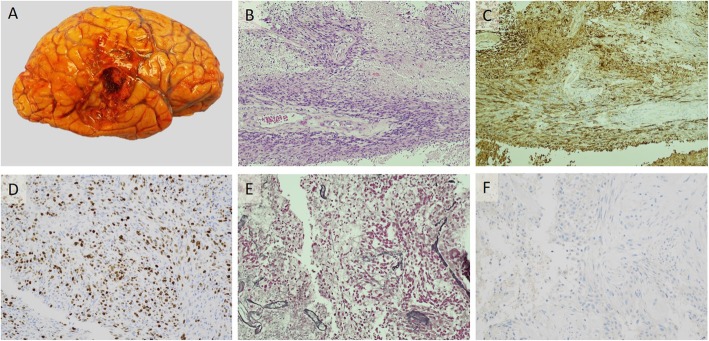
Histopathology of post-treatment recurrence of IDH-wildtype GBM in the temporal lobe.**a** Gross image of the right temporal lobe with resection cavity (6.0 × 3.5 cm) and residual tumor. **b** Tumor is histologically compatible with GBM on H&E stain. **c** Tumor cells are diffusely positive on GFAP stain. **d** Tumor cells demonstrate positive MIB-1 focally up to 60%. **e** Tumor cells are negative on reticulin stain. **f** Tumor cells are negative (wild-type) on IDH1 R132H stain. Micrographs are 10X magnification

The patient’s mental status deteriorated as the right temporal recurrence rapidly progressed, and she underwent right temporal craniotomy for debulking of the tumor eleven months after her initial diagnosis of GBM (Fig. 2). Histologically, this secondary tumor was identical to the primary. 2 × 10^5^ freshly dissociated cells from the right temporal recurrence were orthotopically transplanted directly into the striatum of SCID mice with preserved microglial activity (IcrTac:ICR-*Prkdc*^*scid*^ strain) to assess the cells’ ability to generate a patient-derived xenograft (PDX). Following surgery, the patient stabilized neurologically, but opted for palliative care and was transferred to hospice where she expired one month later.

**Fig. 3:**
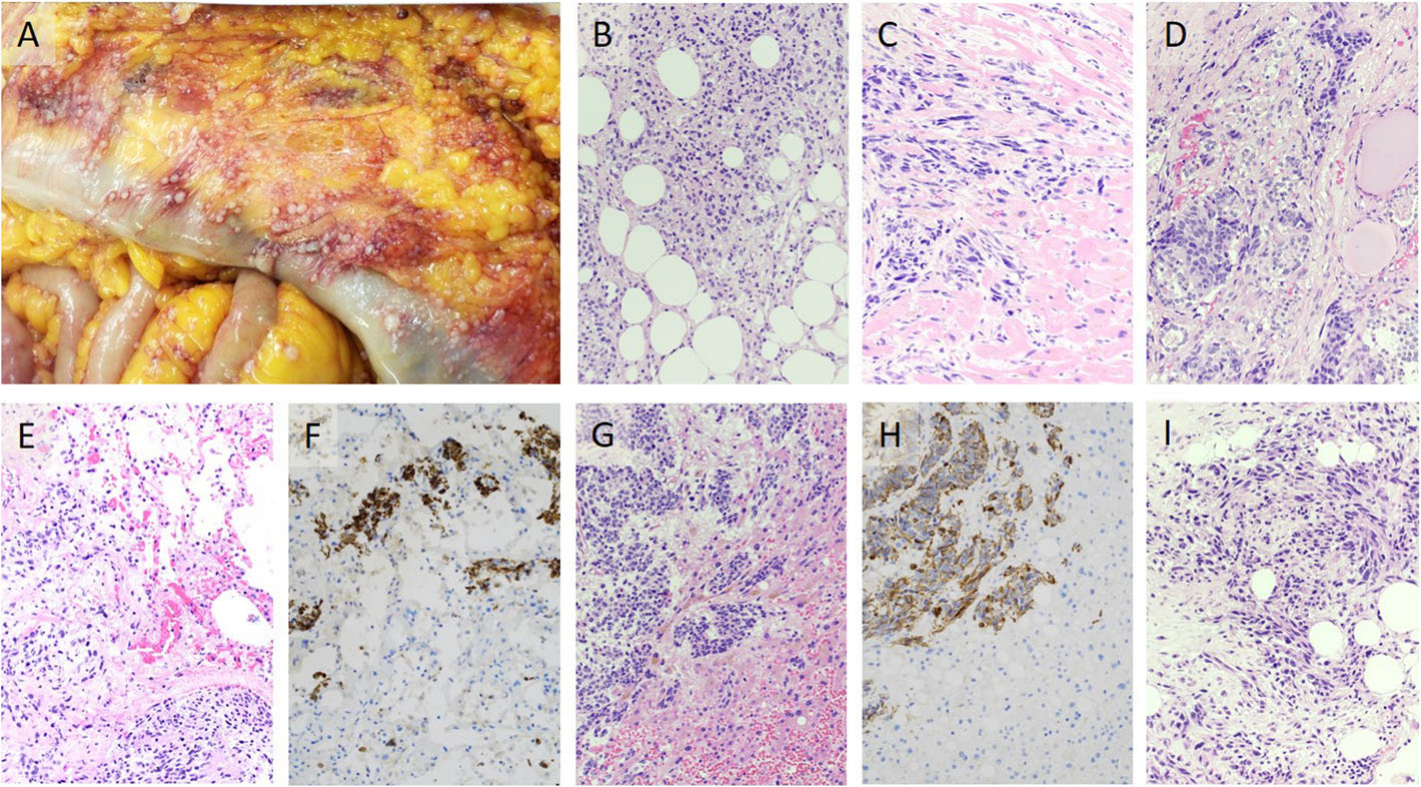
Metastatic GBM lesions involving multiple organs. **a** Gross photo of “peritoneal glioblastomatosis,” i.e., metastatic GBM studding the surface of the small bowel and omentum. **b** Metastatic GBM infiltrating breast tissue (H&E stain). **c** Metastatic GBM infiltrating cardiac muscle (H&E stain). **d** Metastatic GBM infiltrating thyroid parenchyma (H&E stain). Metastatic GBM infiltrating lung tissue (**e** H&E stain, and **f** GFAP stain). Metastatic GBM infiltrating breast tissue (**g** H&E stain, and **h** GFAP stain). **i** Metastatic GBM infiltrating omental tissue (H&E stain). Micrographs are 10X magnification

While the patient was alive, consent was obtained for a rapid autopsy, which was ultimately performed within four hours of death. Gross and histological evaluation confirmed numerous GBM metastases. The extent of metastatic disease was widespread, including bilateral lungs, heart, liver, thyroid, left breast, small bowel, omentum, peritoneum, left pelvic bone, and hilar lymph nodes (Fig. 3). Notably, there were extensive metastatic lesions involving the abdominal cavity.

The autopsy revealed residual GBM in the original site of occurrence (left occipital lobe). On histological examination of pre- and post-mortem samples, the metastases appeared identical to the primary tumor and temporal recurrence. All sites demonstrated the classic appearance of GBM on H&E staining, including nuclear atypia, microvascular proliferation, and pseudopalisading necrosis. Sarcomatous transformation was not identified on histological review of the tissue sections, and was confirmed absent by reticulin-staining performed on representative sections from occipital lobe, temporal lobe (Fig. 2e), left breast, heart, lung, liver, lymph nodes, and omentum.

To evaluate the molecular phenotype of the primary, recurrent, and metastatic lesions, two different next-generation-sequencing (NGS) panels were employed (see Additional file 1). The Ion AmpliSeq Hotspot Cancer NGS Panel v2, covering 50 genes and 207 amplicons, was performed on all pre-mortem (left occipital brain primary, right temporal lobe recurrence, and left breast metastases) and select post-mortem (paratracheal lymph node and omentum) tissue specimens. The Ion Torrent Oncomine Comprehensive Assay v3, covering 161 cancer driver genes, was performed on the remaining post-mortem samples (left occipital lobe, right temporal lobe, left breast, lungs, and liver) collected during rapid autopsy.

**Fig. 4:**
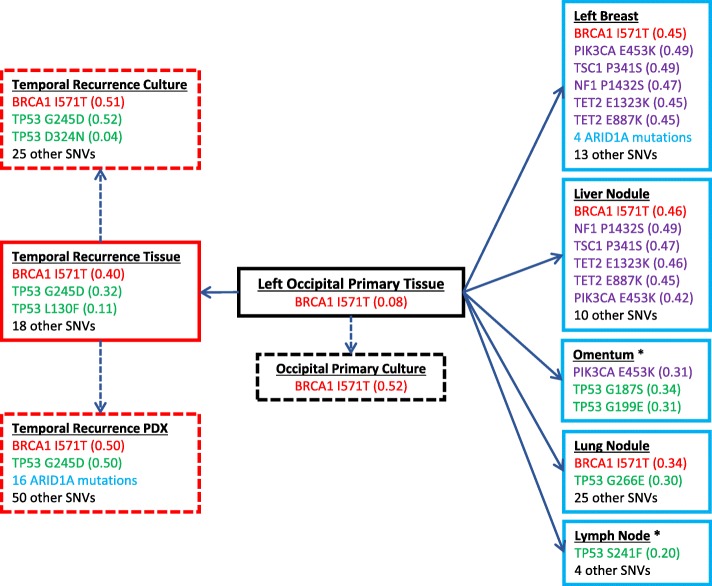
Comparative analysis of coding SNVs identified in metastatic GBM tissue and derived cultures. *BRCA1* p.I571T, an early putative driver mutation found at all time points -- primary resection (black boxes), brain recurrence (red boxes), extracranial metastatic sites (blue boxes) -- is highlighted in red text. Several distinct *TP53* SNVs (green text) were identified in the temporal recurrence and multiple metastatic sites, suggesting convergent evolution. Several SNVs (purple text) were shared between metastatic sites, indicating a common ancestral clone. To assist in distinguishing putative driver and passenger mutations, variant frequencies (in brackets) were compared between source tissue (solid boxes) and cultured or xenografted cells (dashed boxes). Asterisks indicate samples sequenced using Ampliseq Cancer Hotspot panel only

The AmpliSeq panel revealed single nucleotide variants (SNVs) in *PIK3CA*, *SMARCB1*, *BRAF*, and *TP53*. Notably, five different SNVs were detected for *TP53*. There were differences in *TP53* mutations between the primary tumor, metastases, and among the metastases themselves (Fig. 4). The more comprehensive Oncomine panel revealed only one non-silent SNV common to all specimens: *BRCA1* p.I571T. A large number of private mutations were detected in the temporal lobe recurrence, left breast, lung, and liver metastases. Among the extracranial metastatic sites, only the left breast, liver, and omentum specimens were found to share any mutations other than the one identified at *BRCA1*.

**Fig. 5:**
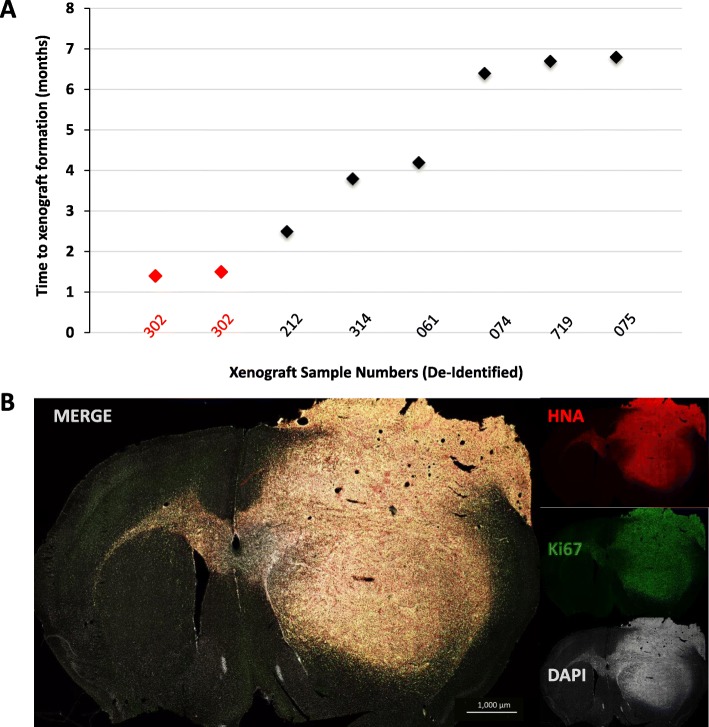
Patient-derived xenograft of recurrent temporal lobe GBM in a SCID mouse brain. **a** Time to fatal xenograft formation for cells derived from the index case (sample ID 302) versus six other consecutive cases of IDH-wildtype GBM not associated with extracranial metastasis. **b** Micrograph of mouse brain 5 weeks after xenotransplantation into the right striatum of 200,000 GBM cells obtained during resection of the temporal lobe recurrence. Tumor cells are seen infiltrating the contralateral hemisphere via the corpus callosum. HNA, human nuclear antigen. Scale bar = 1000 μm.

Cells cultured from the left occipital primary site at the time of initial diagnosis, and the right temporal site at the time of recurrence, expanded readily in serum-free conditions and were all found to harbor the *BRCA1* p.I571T mutation (see Additional file 1). Both mice, orthotopically xenotransplanted with cells from the temporal recurrence, developed fatal malignant gliomas after 5 weeks, confirming the tumor’s aggressive behavior in both human and rodents (Fig. 5). Necropsy analysis of the lungs, intestines, liver, and spleen in these mice did not reveal the presence of any peripheral metastases as seen in the patient. Tumor cells from this aggressive GBM were isolated from the primary PDX and were subsequently propagated in culture and used to generate a reliable PDX model which forms within 3–4 weeks post implantation and, importantly, recapitulates both the rapid growth and the malignant infiltrative spread of human GBM. Mutational hotspot analysis of the PDX confirmed the presence *BRCA1* p.I571T in all cells, and multiple inactivating mutations of *ARID1A* in a significant subpopulation. ARID1A mutations were also identified in the left breast autopsy specimen (Fig. 4).

**Fig. 6:**
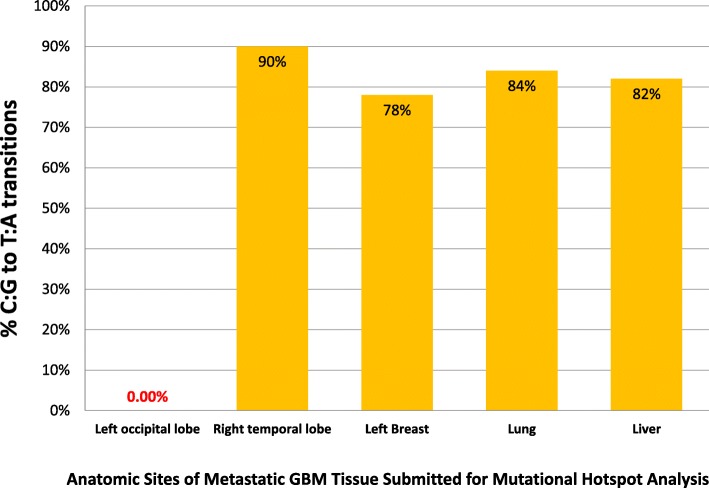
Percentage of all somatic SNVs identified through targeted sequencing (Oncomine Comprehensive Assay version 2) of primary, recurrent, and metastatic GBM specimens that were C:G to T:A transitions

To investigate whether the relatively high number of SNVs in all secondary sites compared to the primary could have resulted from mismatch repair (MMR) deficiency, we performed a mutational spectrum analysis; results confirmed a high proportion of C:G to T:A transitions, which is typical of the mismatch repair deficiency described in the setting of temozolomide-treated recurrent GBM (Fig. 6). The integrity of the mismatch repair pathway was evaluated using immunohistochemistry. All metastatic specimens exhibited positive staining for anti-MLH1, anti-MSH2, anti-MSH6, and anti-PMS2, indicating microsatellite stability (MSS). MSS was further confirmed with PCR of established microsatellite loci [17] (see Additional file 1). In addition, we evaluated DNA polymerase epsilon (*POLE*) using PCR, which showed no alterations at mutational hotspots within the coding region of the gene.

## Discussion and conclusions

GBM is known to have aggressive biological behavior with poor survival outcomes [1], as demonstrated in this case of a woman who expired approximately one year after initial diagnosis. At autopsy, in addition to the widespread extracranial metastases, this case had a unique gross finding. In the abdominal cavity, metastatic lesions extensively studded the surfaces of the small bowel, omentum, and peritoneum, mimicking the appearance of peritoneal carcinomatosis; therefore, when secondary to GBM, we propose the term “peritoneal glioblastomatosis” to describe this rare presentation (Fig. 3a).

While the molecular variants associated with GBM and its subtypes are well-described [18, 19], there remains a critical gap in understanding which genomic drivers may lead GBM to metastasize. The unusually large number of private SNVs observed in all specimens except the left-occipital primary suggests that a parental clone at the primary site acquired a hypermutator-like phenotype during adjuvant chemoradiation and subsequently seeded the extra-CNS sites, possibly via invasion of the sagittal sinus. The hypermutating subclone also seeded the contralateral temporal lobe – presumably via white matter migration – and was able to expand due to its location outside the primary radiation treatment volume. GBM dissemination via CSF pathways is also a recognized possibility, but in this case less likely to have been a major mechanism given the absence of diffuse leptomeningeal disease or spinal drop metastases.

Gliosarcoma is a rare variant of GBM with an increased tendency to metastasize [20], and must be considered in the differential diagnosis in this case. In gliosarcoma, metaplastic transformation of gliomatous tumor gives rise to a sarcomatous component, which is associated with a higher rate of connective tissue invasion and extracranial metastasis [20]. This transformation has been linked to the acquisition of driving *TP53* mutations [21]. In our case, sarcomatous histology was not identified in any of the primary or secondary specimens; thus, the pathogenetic mechanism of extracranial spread of GBM in our case is likely distinct from that seen in gliosarcoma. Although numerous *TP53* mutations were detected, none were shared between sites, suggesting they arose as a product of genetic instability in a parental clone, rather than as primary drivers of the instability. Consistent with this view, Park et al. detected multiple different *TP53* mutations between sites in 2 out of a series of 6 metastatic GBM cases examined. They suggest that this resulted from dissemination of subclones that were dormant in the primary tumor, which then activated and expanded in the metastatic microenvironment [16].

Since it was the only coding alteration common to the primary tumor, recurrence, and metastases, we consider the *BRCA1* p.I571T SNV to be the most likely driver of this GBM’s unique metastatic phenotype. Although there is no literature to date describing a role for *BRCA1* mutations in GBM pathogenesis, alterations in *BRCA2* have been associated with genomic instability in astrocytomas [22], and a *BRCA2* inactivating mutation was found in the primary site of a metastatic GBM [23]. Perhaps screening for metastatic disease may be considered when BRCA mutations are found in a primary GBM. Among GBM specimens in The Cancer Genome Atlas (TCGA), *BRCA1* and *BRCA2* missense mutations are rare, each occurring at a rate of 1.4%. Piccirilli et al. [24] described a series of 11 patients with a history of invasive breast carcinoma who subsequently developed GBM; however, an analysis of *BRCA1* or *BRCA2* mutational status was not performed. *BRCA1* defects are known to dysregulate cell checkpoint pathways and impair the fidelity of the DNA damage response, particularly to double-strand breaks (DSBs) [25]. We speculate that GBM cells with *BRCA1* defects might exhibit particularly high levels of genomic instability when exposed to DSB-inducing agents such as RT and temozolomide, increasing the risk of treatment-induced cancer evolution and acquiring new, aggressive phenotypes.

In GBM and other solid malignancies, a high proportion of acquired C:G to T:A transitions is classically associated with chronic exposure to alkylating agents in the context of a deficiency in one or more components of the DNA mismatch repair machinery. Possible mechanisms include acquired inactivating mutations or epigenetic silencing of the MMR genes *MSH6*, *MSH2*, *MLH1*, and *PMS2*. Recent studies show that treatment with TMZ of *MGMT* unmethylated tumors, such as in our case, introduces a strong selective pressure to lose mismatch repair pathway function [26]. Although immunostaining demonstrated intact MMR protein expression in the recurrent and metastatic specimens of our case, mutational hotspot analysis of the PDX derived from the temporal recurrence revealed inactivation of ARID1A, which has recently been shown to promote MMR by interacting with MSH2 [27]. *ARID1A* mutations are rare in GBM, occurring at a rate of 0.7% in newly diagnosed cases, and may be associated with an aggressive phenotype. Both cases described in TCGA were seen in males under the age of 50, one of whom survived less than 1 year. Thus, our case illustrates the need for caution in the treatment of MGMT unmethylated GBM with TMZ, even if the tumor exhibits microsatellite stability by conventional methods, since other forms of instability may exist.

In considering alternatives to TMZ, new data suggests that PARP inhibitor therapy may be effective in ARID1A- as well as BRCA-defective tumors [28]. ARID1A-defective tumors may also be particularly good candidates for immune checkpoint blockade due to the potentially large number of immune-activating neoepitopes generated by MMR deficiency [27]. The PARP inhibitors olaparib and BGB-290, among others, are currently being evaluated as radio- and chemosensitizers in both IDH-wildtype and IDH-mutant GBM in early phase clinical trials, but no molecular biomarkers for response have so far emerged [29–31].

In conclusion, we describe a rare and highly aggressive case of widely metastatic IDH-wildtype GBM with a clonal somatic mutation in *BRCA1*. Post-treatment recurrent tumor in the brain and in multiple systemic organs exhibited evidence of acquired DNA mismatch repair deficiency, despite retaining intact expression of mismatch repair pathway proteins. This may be explained by loss of ARID1A, which is required for MSH2 function.

## Supplementary information

**Additional file 1.** Supplementary Methods.

## Data Availability

The data that support the findings of this study are not publicly available to protect patient privacy, but are available from the corresponding author upon reasonable request.

## Abbreviations

GBM: Glioblastoma
MMR: Mismatch repair
MSS: Microsatellite stability
NGS: Next-generation sequencing
PDX: Patient-derived xenograft
SNV: Single nucleotide variant
TCGA: The Cancer Genome Atlas
TMZ: Temozolomide
