# Supplementary material for: Widely metastatic glioblastoma with BRCA1 and ARID1A mutations: a case report
Source: BMC Cancer. 2020 Jan 20;20:47. doi: 10.1186/s12885-020-6540-1 (PMC6971940; doi:10.1186/s12885-020-6540-1)
Supplement: Supplementary file 1 — Additional file 1. Supplementary Methods. [file 12885_2020_6540_MOESM1_ESM.docx]

**Supplementary Methods**

Cell Culture

All GBM cell lines used to generate cell cultures in this study were derived from patients enrolled in our institutional IRB-approved cancer biorepository tissue procurement protocol. En bloc resected tumor tissue was first processed for clinical diagnosis according to clinical standard operating procedures. To establish cultures, freshly resected tumor tissue was washed and digested as previously described [1] . After centrifugation, cells were resuspended in 6 mL NeuroCult NS-A Proliferation Medium (Stemcell) supplemented with recombinant human EGF (20 ng/mL), and 0.0002% heparin, then cultured in T25 tissue culture flasks. The medium was changed at 48 hours and then every 3-4 days. Once red blood cells and other debris were cleared from the cultures and a phenotypically homogeneous population of cells began to expand, DNA, RNA and protein were collected every five passages for molecular characterization. At each passage, cell counts were tracked to determine growth kinetics. All experiments were performed at passage 20 or lower.

Assessment of Microsatellite Instability

To determine MSI status, genomic DNA was isolated from GBM tissue specimens and primary culture cells using PureLink Genomic DNA Mini Kit (Invitrogen, K1820-01) according to the manufacturer’s instructions. Genomic DNA isolated from peripheral blood mononuclear cells (PBMC) was used as the control. 50 ng of each genomic DNA sample was amplified with five mononucleotide markers/primers (BAT-25, BAT-26, NR-21, NR-22 and NR-24) as described in a previous study [2] using TaKaRa rTaq DNA Polymerase (Takara, R001AM). MSI status was evaluated by electrophoresis. 40% Acrylamide/Bis (19:1) was used to pour the gels. All amplicons generated from GBM tissue specimen DNA were run adjacently to those generated from PBMC DNA. Specimens that exhibited size variability in more than 3 MSI markers were considered MSI-high, as per previously established criteria [2].

Targeted Next-Generation Sequencing

The Oncomine Comprehensive Assay version 3 (OCP v3, Thermo, #A35805) was used as previously described [17] to perform targeted next-generation sequencing. Analyses of raw sequencing data were performed with Ion Torrent Suite (version 5.0.2) using the “coverageanalysis” and “variantcaller” plugins as per the manufacturer’s guidelines. The “variantcaller” output on each bulk and cultured sample was filtered to exclude variants occurring at a frequency of less than 0.05. These were further filtered to exclude identical variants occurring in the matched PBMC DNA sample. The functional significance of variants was assessed using mutationassessor.org. All somatic coding and non-coding variants were considered when calculating the percentage of CG>TA transitions.

**References**

1. Pain M, Wang H, Lee E et al. Treatment-associated TP53 DNA-binding domain missense mutations in the pathogenesis of secondary gliosarcoma. Oncotarget. 2018; 9(2): 2603-2621.

2. Suraweera, N. et al. Evaluation of tumor microsatellite instability using five quasimonomorphic mononucleotide repeats and pentaplex PCR.  Gastroenterol. 2002; 123(6):1804-1811.
